# Opium

**Published:** 1853-03

**Authors:** 


### ART. III. — *Opium.* The opinions thereon of Schrappenkuttel Smelfungus, M. D.

"I acknowledge no Procrustean creed, decapitating nonconformity!"

All is blue in the office of Smelfungus. He is blowing a cloud, and the room is filled with the aroma of tobacco, about the paternity of which there can be no doubt — it is genuine Connecticut, and no mistake.

"Longevity," he says, "cannot be considered a characteristic of the materia medica. From the earliest days of medicine, ephemeræ have arisen; fluttered out their existence, and disappeared from the view. But other articles there are, which possess inherent vitality, which have been for ages the main pillars of therapeutics; and which still stand firm in their stately proportions. And stateliest, with firmest shaft of these, is opium.

"Opium! mysterious drug, whose potency is felt not only in the body, but in the recesses of the mind: where is the link that connects the sleepy poppy with the grandest powers of our nature? By what deep-hidden agency does it lull the racked nerve to quiet, and steal upon the mind with gorgeous dreams, extending time and space beyond conception? No man has yet returned from these close penetralia with power to tell their secrets. Used daily and hourly for many centuries, it is still unknown, misunderstood, abused, and underrated. De Quincey, wrapped in Elysian dreams by its still influence, quaffing by imperial pints his 'happiness in bottles,' with powers of utterance never equaled, 'speaking as never man spake,' has lifted the silver vail, only to reveal behind it the blackness of darkness. He has called from out the depths; but his voice is choked by sorrow, and we hear sighs only, — *suspira de profundis.* Oh mighty agent! instrument by which the soul dips down to Acheron, and gazes through the portals of Tartarus; no power can interpret or destroy thee!"

"Now, Smelfungus," I venture to insinuate, "you are on stilts as long as that euphonious baptismal name of yours, and allow me to suggest, you are gyrating rather awkwardly. How are you going to get down?"

Smelfungus looks grieved, holds silence for a moment, and then abruptly changes his style of address.

"Have you noticed, my friend, how all things work together for good to them that love physic? Have you seen how, out of this expectant humbug, have come goodly things, figs from thistles, grapes from thorns? Our over-wise Expectant must needs do something. His lazy theory was to wait for positive indications; and very naturally, one eternally recurring indication was the relief of pain. So our friend Expectant follows it out, and when you come to examine his treatment, you find here, there, everywhere, opium, and opium alone, prescribed in all the ills that flesh is heir to, from Abscess to Unknown. Of course this was nonsense and error. Better and more scientific was old Dr. G.'s prescription for a 'singing in the head," viz., a poultice of old music books applied to the coccygeal region, with the luminous idea of 'drawing the music down!' There's revulsive treatment for you! But after all, good cometh from every new thing. Some of Expectant's patients get well, and that without regard to old time depurative theories. And some of these were cases whereof Dame Nature had not the handling. Dame Nature is a botch! To relieve a pleuritic inflammation, she fills one side of the chest with water; a remedy from which the patient might well pray to be delivered. But Expectant coming to a case of incipient pleuritis, makes a homœopathic diagnosis, calls it 'pain in the side,' and gives three or four grains of opium therefor. Patient gets well instanter. Well, Expectant comes to another patient, finds her with low typhoid symptoms, and abdominal tenderness, but not much else to complain of. In the absence of any decided indications, Expectant prescribes his eternal opium. By-and-by this patient, too, recovers, and Mr. Observer, standing by, beholds a case of puerperal peritonitis cured by opium.

"From all this, I, sir, sitting in philosophic judgment, derive the great fact that we know nothing about opium. Tell us, old Monument, who have for forty years dealt out your opium, what know you about it? Did you ever dream that it was a *curative* you were handling, and not a palliative merely? Man of the microscope and testing tube, read for us this deep, this Sphynx-like riddle. Are we yet — we staunch old regulars — to yield the field in this matter? Is opium the curative means, the efficient drug in our mixed prescriptions of calomel et opii. $\overline{aa}$ grs. iij? For look you, country practitioner dealing Schieffelin's best powdered from the blunt point of your old jack-knife, you old Clysterpipe, who have not weighed a prescription these half-score years, your small doses of opium weigh three stout grains!

"*Rest!* That is the word. Here, in rest and sleep, 'tired nature's sweet restorer' lies the secret. One sensible thing that Dame Nature does, is to put her patients to bed — to so prostrate their lusty muscles, that the sturdy knees give way, and the recumbent posture becomes necessary. Here, then, is the great curative, and opium is a means thereto. 'Old Functional Harmony' used to tell us with extremest unction, that 'uterine contraction was the remedy for uterine hæmorrhage.' Not lead, not ergot, not cold water, not compression, not the tampon, but any one, or all of these, which would bring on uterine contraction. So here in inflammation. Rest is our remedy — not calomel, not antimony, not bloodletting, not opium, but any one, or all, or none of these, so that you secure rest.

"There is a vague idea, derived from some exploded theories, that calomel has, *jure divino,* a certain control over inflammation — that the presence of calomel in the stomach, simultaneously with inflammation in the viscera, is incompatible. Now, good Sir Hunker! you know that I — Smelfungus — have the highest respect for you of the conservative school. You know that on every occasion I have ranked myself among you — have bowed to the existence of a liver — and scouted at the pretensions of these new comers, who bear that flaunting 'banner with the strange device,' *Young Physic.* But, my dear sir, we must compromise or surrender. Let us come down a peg or two and we shall still be men of note. Let us use a little Twigg's hair dye, and rejuvenate ourselves — gray hairs are at a discount now-a-days. Look you! only a day or two since a certain divine declared a decided preference for young physicians, over old. He had the hardihood to intimate that one good theory, sound and well established, was worth ten years' experience. He was a Scotchman, and I said to him, that 'it behoveth a Scotchman to be right, for if he be wrong he be forever and eternally wrong.' Between you and I, my aged friend, it is about time to cave. Now I have a talent for compromises, and I can propose a satisfactory arrangement which shall govern this vexed question of Calomel *vs.* Opium. Let us hereafter say nothing about being 'bilious.' That's all well enough at the bedside, but it is ruled out of professional intercourse. Let us give our calomel as we did of old to all patients of firm fibrinous habits, whose blood has a tendency to plastic exudation. So much we claim for our side of the house. Now we may as well, (needs must when the devil drives,) concede to Young Physic, that calomel shall be withheld in cases where this condition does not obtain, viz., in those manifold diseases where the blood has a tendency to fluidity. I fear that this will narrow down the amount of the drug used, but we must come to it. Do n't you recollect feeding calomel, for a fortnight on a stretch, to that strumous little girl with dysentery, last year? How fast she did gain, did n't she? And how nicely you could trace the curative influence of the calomel, could n't you?" And Smelfungus puts his tongue in his cheek, and makes a mysterious gesture with his thumb over the left shoulder. And I can imagine Editor Flint perusing this backsliding confession of Smelfungus with a quiet smile and a chuckle, which means "I told you so!" But Smelfungus loves freedom of action; he cannot bear to be fettered by the green withes of tradition. Witness the motto at the head of this article.

But touch him gently, Dr. Flint! or you may yet see Smelfungus astride his old bilious Rosinante, charging the windmills of natural medicine with the stern voiced war cry, "*Floret Calomelas — ruat coelum!*"

> "Salts, sir, in all his steps — manna in his eye —
> In every gesture, calomel and rhubarb."

Smelfungus loves fun, and from no recent occurrence has he derived so many good horse-laughs as from the developments at Bellevue *in re* of opium in puerperal peritonitis.

"Dr. Clark would have made no great stir with his interesting experiments in peritonitis, had he not been so severely criticised. Not but that Dr. Clark's treatment was a goodly instance of *a priori* reasoning applied to therapeutics — brilliant in its conception, and triumphant in its success. But the fun of the matter lies in the criticism of the New York Medical Gazette. 'This,' he says, 'comes of making hospital doctors of mere theorists.' He tells us that we must look in the dead-house records for the results of such treatment. *Of course* the patients are dead. He stops not to inquire about it — they are dead *de necessitate;* and he sheds his tears over them as freely as a child in the measles. Smelfungus can see him; leading a lachrymose group of anxious inquirers beside the green shores and still waters of the East River. With solemn step and slow, he conducts them down the gravel walks of the garden at Bellevue, 'twixt cabbages and onion beds, and sadly points to the little dead-house, as filled with the sad mementoes of Dr. Clark's recklessness.

"But they look in vain — these women are not yet defunct, but still live to bend as best they may, with abdomen probulgent over their wash tubs, the spared monuments of human folly."

"Oh that mine enemy had written a book!"

If I were to tell you, gentle reader, all that Smelfungus says about this matter, I should detract from that solemnity which becomes the pages of a medical journal. A pompous dignity is the main-stay of our profession, and by a parity of reasoning a medical monthly should indulge in no unseemly cachinnations. *Vale.*

